# Proteome-wide profiling and mapping of post translational modifications in human hearts

**DOI:** 10.1038/s41598-021-81986-y

**Published:** 2021-01-26

**Authors:** Navratan Bagwan, Henrik H. El Ali, Alicia Lundby

**Affiliations:** 1 Department of Biomedical Sciences, Faculty of Health and Medical Sciences, University of Copenhagen, 2200 Copenhagen, Denmark; 2 The Novo Nordisk Foundation Center for Protein Research, Faculty of Health and Medical Sciences, University of Copenahagen, Blegdamsvej 3, 2200 Copenhagen N, Denmark

**Keywords:** Protein analysis, Proteome informatics, Proteomics, Mass spectrometry, Peptides, Proteomics, Post-translational modifications, Acetylation, Glycosylation, Methylation, Neddylation, Nitrosylation, Phosphorylation, Prenylation, Ubiquitylation

## Abstract

Post translational modifications (PTMs) are covalent modifications of proteins that can range from small chemical modifications to addition of entire proteins. PTMs contribute to regulation of protein function and thereby greatly increase the functional diversity of the proteome. In the heart, a few well-studied PTMs, such as phosphorylation and glycosylation, are known to play essential roles for cardiac function. Yet, only a fraction of the ~ 300 known PTMs have been studied in a cardiac context. Here we investigated the proteome-wide map of PTMs present in human hearts by utilizing high-resolution mass spectrometry measurements and a suite of PTM identification algorithms. Our approach led to identification of more than 150 different PTMs across three of the chambers in human hearts. This finding underscores that decoration of cardiac proteins by PTMs is much more diverse than hitherto appreciated and provides insights in cardiac protein PTMs not yet studied. The results presented serve as a catalogue of which PTMs are present in human hearts and outlines the particular protein and the specific amino acid modified, and thereby provides a detail-rich resource for exploring protein modifications in human hearts beyond the most studied PTMs.

## Introduction

Post translational modifications (PTMs) are recognized as important mechanisms for subtle or dramatic alterations of protein function and provides a mean for cells to regulate diverse molecular processes. PTMs regulate folding, localization, interactions, degradation and activity of proteins and they partake in essentially all biological processes. Due to the rapid and reversible nature of many PTMs they are also ideal mediators in signal transmission. In the heart, PTMs are involved in diverse mechanisms, such as regulation of contraction, heart rate and metabolism. For the fight-or-flight response, protein phosphorylation changes of components of the excitation–contraction coupling process are essential, and early studies confirmed functional regulation of phospholamban, troponin I and the ryanodine receptor by phosphorylation^1–3^, which underscore the importance of this PTM in cardiac physiology^2^. With advances in mass spectrometry based proteomics, studies of protein phosphorylation can be performed in a manner where essentially all phosphorylated proteins are investigated outlining complete signalling pathway regulation in hearts^4^. Despite the notion that PTMs, such as phosphorylation, acetylation, ubiquitination and glycosylation^5–9^, mediate fundamental molecular mechanism in the heart only a small subset of the more than 300 known PTMs have been investigated in heart tissue.

Shotgun mass spectrometry (MS)-based proteomics has become a powerful tool for biomedical research. The sensitivity and speed of MS instruments allow for measurement of millions of spectra and identification of thousands of proteins and peptides in single experiments. A critical component of the analysis of shotgun proteomics datasets is the search engine, which is an algorithm that attempts to identify the peptide sequence corresponding to the measured mass to charge ratios by matching the information in acquired spectra with in-silico generated information from a protein database^10^. In conventional search engines, the number of PTMs that can be queried is restricted to a few at a time. Among the most commonly queried PTMs are phosphorylation on serine, threonine or tyrosine residues and chemical modifications such as oxidation of methionine residues. With restricted search approaches of acquired data, mass shifts produced by any other modification are not considered, and any spectra containing ‘unknown’ mass shifts remain unidentified. This is likely a major contributor to a majority of spectra remaining unidentified in shotgun proteomics experiments.

In recent years, a number of computational methods have been developed aiming at identifying unidentified spectra. These methods count tools as Comet-PTM^11^, MSfragger^12^ and PTMselect^13^, which are broadly known as ‘open searches’ or ‘error tolerant’ search algorithms. The strategy of these approaches are to use precursor mass tolerances of hundreds of Daltons and including any PTM in the fragment spectrum evaluation. These strategies established that many previously unidentified spectra could indeed be matched to peptide sequences when PTMs were included in the spectra evaluations^11,14^. The open search algorithms have been utilized to identify involvement of previously unknown PTMs in diseases^11,12,15^.

Our knowledge of which PTMs are present on proteins in the heart is incomplete and this per se limits our ability to investigate for dysregulation of PTMs in heart disease. To address this, we applied the Comet-PTM search engine and associated tools^11^ to explore which PTMs are present on proteins in the human heart. We classify and characterize PTMs identified in samples from human hearts collected from seven individuals and covering three of the cardiac chambers. Our study presents a catalogue of which PTMs are present on cardiac proteins in human hearts, it outlines the particular protein and the specific amino acid residues that are modified, and thereby provides a detail-rich resource for exploring protein modifications in the human heart well beyond the best known PTMs. This is an essential step prior to studying involvement of novel PTMs in context of cardiac disease.

## Results

### Comprehensive landscape of post translational modifications in cardiac tissue samples

**Figure 1:**
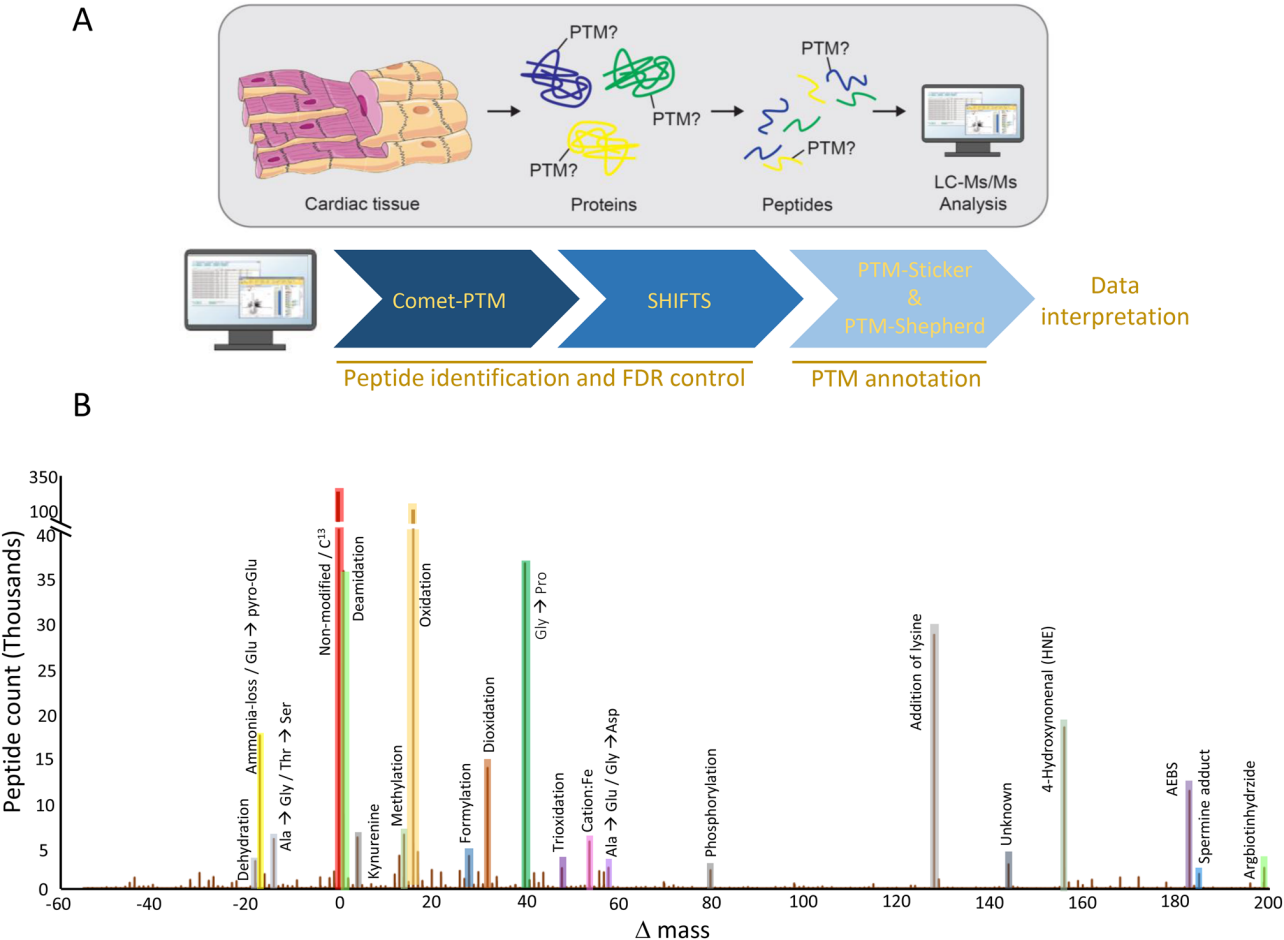
Shotgun proteomics workflow and analysis pipeline used for PTM identifications. (**A**) In the dataset analysed herein, cardiac tissue was homogenized, proteins were extracted from the tissue and enzymatically digested to peptides by trypsin. The peptide mixture was then measured on a high-resolution tandem mass spectrometer from which MS/MS spectra were obtained. These MS/MS spectra were then searched with the Comet-PTM search engine against a human protein database and the results were processed with SHIFTS for PTM peak detection and FDR control. Detected PTM peaks were subsequently annotated by the tools PTM-sticker and PTM-Shepherd. (**B**) Cumulative frequency distribution of Δ masses obtained by the Comet-PTM and SHIFTS analysis of human heart proteome dataset. In the figure, highlighted peaks represent different PTMs identified based on their Δ Mass in the range of − 55 to 200 Dalton. Phosphorylation of a peptide is for instance identified in the tandem mass spectrum as a mass shift of 79.966 Dalton and methylation is identified as mass shift of 14.015.

To explore and determine the variety of post translational modifications present on proteins in the human heart, we investigated a deep proteome dataset from seven individuals covering three of the heart chambers^16^—left atria (LA), right atria (RA) and left ventricle (LV) (Fig. 1A, upper panel)—for post translational modifications. We analyzed the raw files and identified peptides using the search engine Comet-PTM^11^ (Fig. 1A, lower panel). Comet-PTM enables thorough analysis of PTMs and as previously benchmarked^11^ we confirm improved scores for modified peptides by Comet-PTM in this human heart data compared to those obtain in a closed search (Supplementary Fig. S1). Comet-PTM takes the mass shift produced by a modification into account in the fragmentation series and identifies the site of modification by placing the mass shifts (Δ Mass) into the fragmentation spectrum. An example of how various post translational modifications are determined from the Δ Mass distribution is illustrated in Fig. 1B. We processed the output from the Comet-PTM search with SHIFTS^11^, which is an algorithm that detects the peaks in the Δ Mass distribution and controls the peptide false-discovery rate (FDR). The FDR evaluation is performed using a conservative approach, where each modification undergoes an independent FDR estimation to avoid bias created by different proportions of modified and unmodified peptides across samples^11^. Applying a FDR of 0.05 we identified thousands of peaks covering approximately 150 different post translational modifications, which were distributed across approximately 60% of the peptides in the human heart samples. For a complete list of all modifications identified, see Supplementary Table S1.

The modifications identified include PTMs of smaller molecular weight such as methylation (Δ Mass: 14.015 Da), oxidation (Δ Mass: 15.994 Da) and kynurenine (oxidative modification of tryptophan, Δ Mass: 3.994 Da) as well as larger molecular weight modifications such as phosphorylation (Δ Mass: 79.966 Da) and gluconoylation (Δ Mass: 178.045 Da). We also detected rare and more complex modifications such as glycosylation. Of the modifications found, protein phosphorylation and acetylation are among the most studied, and they play important roles in cardiac signalling^4^ and disease states^2^. Herein, we identified thousands of phosphorylated and acetylated peptides, where we assigned the modification to a particular amino acid (see Supplementary Tables S2–S4 for details. For left atrium (S2), for left ventricle (S3), and for right atrium (S4), respectively). Hundreds of these sites have been reported previously confirming solidity of the results. We have included information in the supplementary tables of all phosphorylation-, acetylation- and methylation sites that have been previously reported. Yet, as evident from the supplementary tables, the vast majority of modifications we report are novel.

For all identified PTMs we queried the subcellular localization of the modified protein by utilizing the Compartment Database resource^17^. We have included information on the protein subcellular localization for all PTMs reported across the cardiac chambers (Supplementary Tables S2–S4). Using this suit of algorithms we thus report ~ 150 different protein modifications in human heart samples, which underscores the broad spectrum of post translational modifications of proteins in the heart.

### Distribution of post translational modifications across amino acid

**Figure 2:**
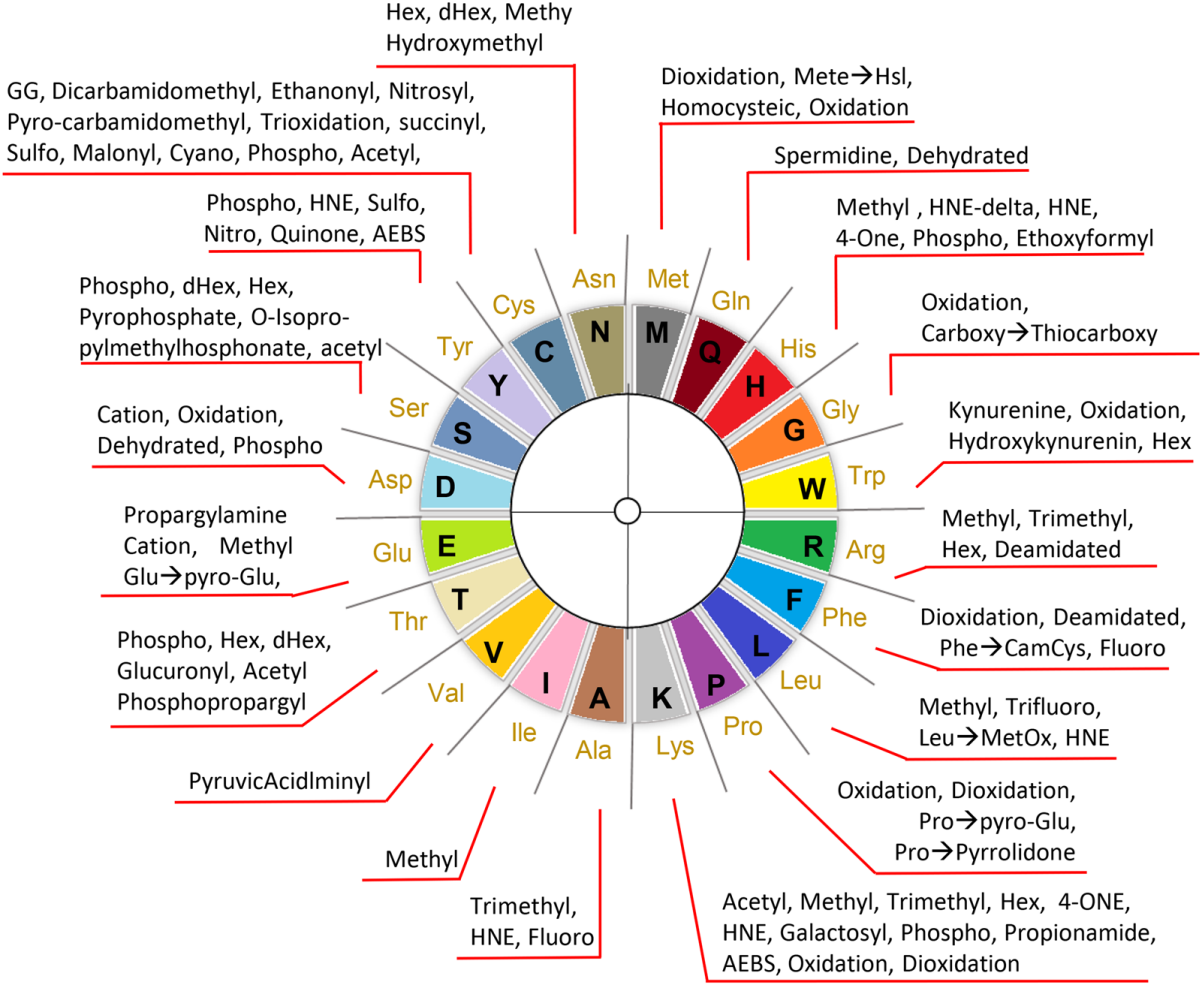
Representation of the post translational modifications identified in our study and their distribution across amino acids. Circular representation of the twenty amino acids and information on the post translational modifications identified on each amino acid. The PTMs were identified in human heart tissues from three chambers (RA, LA, LV) and are represented post processing and FDR control at 0.05. For abbreviations of PTMs see Supplementary Table S5.

The majority of post translational modifications occur on specific amino acids, and the majority of amino acids can harbour different types of modifications. In conventional studies there would be a focused analysis of one or a few post translational modifications at a time, and they would be restricted to specific amino acids. In open searches, using search engines such as Comet-PTM, protein modifications are not restricted to any particular amino acid. By applying an open search strategy, we identified modifications on all 20 amino acids in human heart samples (Fig. 2). As illustrated in Fig. 2, we primarily identify protein phosphorylation on serine, threonine and tyrosine residues, as one would expect^16^. For acetylation, we primarily identify the modification on lysine residues^18^, where it is known to play a significant role in metabolism regulation^19,20^. We also identified acetylation on cysteine, serine and histidine residues, which has also previously been reported, but may be due to non-enzymatic processes^21^. The data summary shown in Fig. 2 illustrates that the biologically important modifications are distributes across all amino acids on proteins in the human heart.

### Classification of identified peptide modifications

**Figure 3:**
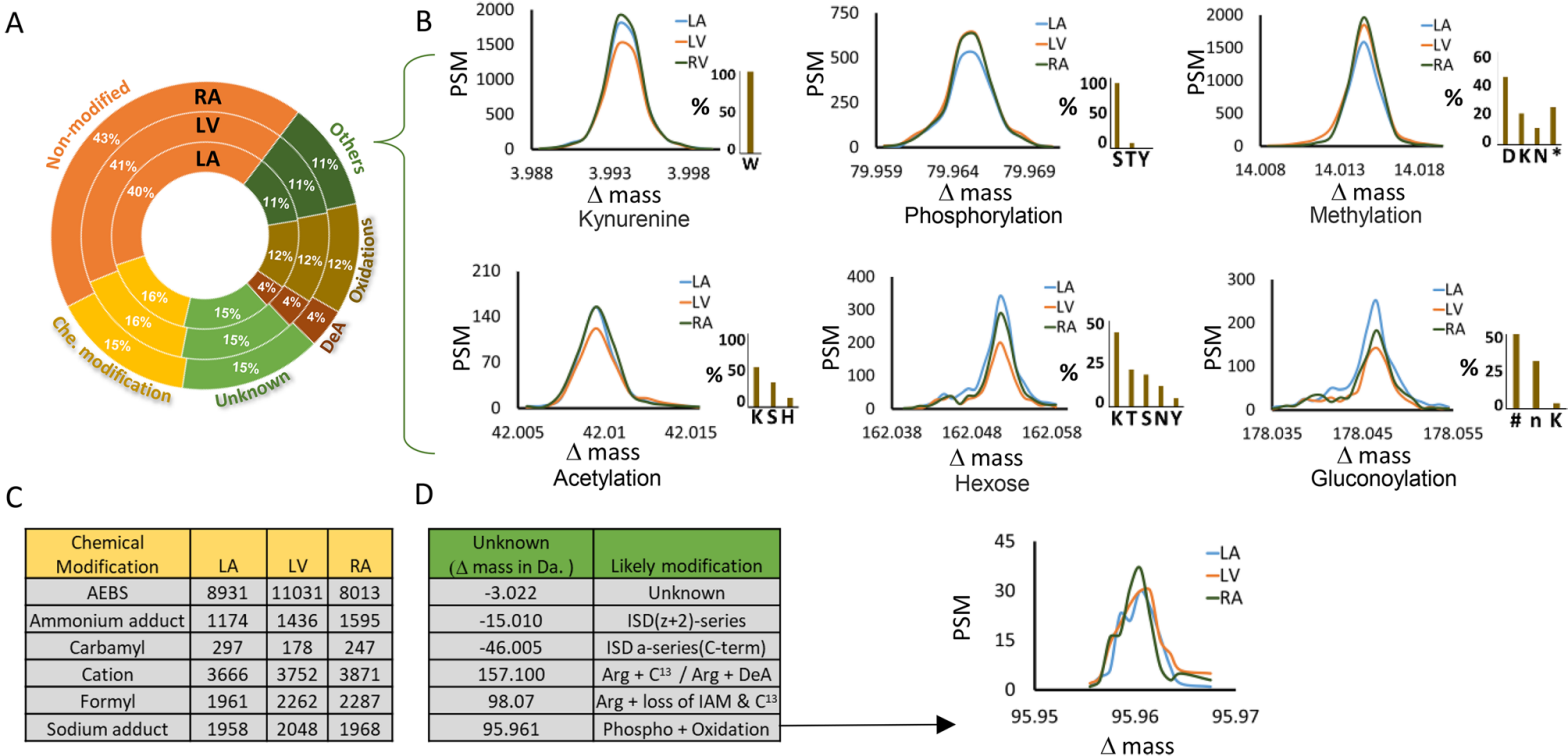
Classification of peptide modifications across three cardiac chambers. (**A**) Pie chart representation of the distribution of identified peptides that were either without a modification (Non-modified), with a chemical modification (Che. modification), a post translational modification (Others, Oxidations, DeA) or with an unknown modification (Unknown). Percentage distributions are shown for each of the three heart chambers (Circular bar graphs: RA, LV, LA). (**B**) Frequency distributions of the occurrence of six biological modifications across the three cardiac chambers. The modifications shown are: Kynurenine, Phosphorylation, Methylation, Acetylation, Hexose/Glycosylation and Gluconoylation. Next to each frequency plot, a bar graph shows the amino acids affected by the modification in percentage. (**C**) Table showing the most common chemical modifications identified in the dataset and the number of peptides carrying the modifications in each of the three chambers of heart. (**D**) Table showing the most frequent unknown mass shifts in the dataset along with a likely explanation of them from PTM-Sticker and PTM-Shepherd. To the right of the table, a frequency plot for the mass shift explained by combination phosphorylation and oxidation is shown.

We next sought to evaluate the distribution of the various modifications across the three regions of the heart that we have analysed (RA, LA, LV). Accordingly, we classified all modifications into categories: known post translational modification, chemical modification and unknown modification. Approximately 40% of the peptides measured across the three cardiac chambers did not carry any modifications (Fig. 3A, ‘non-modified’). This reflects consistency in terms of reproducibility of both the experimental sample processing and the search engine performance. Approximately one third of all peptides measured carried a known post translational modification (Fig. 3A, ‘Others’, ‘DeA’, ‘Oxidations’). Of these, a major proportion were peptide oxidation (12%) and peptide deamidation (DeA, 4%). 11% of all peptides carried other known post translational modifications. These 11% cover tens of different post translational modifications, such as phosphorylation, acetylation, tryptophan kynurenine, methylation, hexose and gluconoylation (Fig. 3B). Reassuringly, all tryptophan kynurenines were localized on tryptophans, and phosphorylations were predominantly found on serine residues with less than 5% localizing to threonine or tyrosine residues, as expected according to literature^22^. Methylation were localized to aspartate or lysine in approximately 70% of the peptides and on serine, threonine or histidine in the remaining cases. This illustrates the precise localization capabilities of the search engine. For examples of manual annotations of fragmentation spectra confirming PTM localizations, see Supplementary Fig. S2.

We identified thousands of peptides with labile modifications such as Hexose (Glycosylation). Being able to identify this PTM simply by an open search strategy is appealing, because glycosylation is involved in cardiovascular pathophysiological processes^23–25^. Labile modifications like hexose are difficult to detect due to the presence of labile glycosidic bond and mostly remain undetected due to ion suppression^26,27^. With ionization techniques such electron capture dissociation or high-energy collision dissociation, as used for the data acquisition of the dataset analysed herein, site mapping is possible. We have primarily detected glycosylation on lysine and threonine residues and to a smaller extend on serine, tyrosine and asparagine residues (Fig. 3B). Another interesting labile modification we observed was gluconoylation^28^, which mainly localized to lysine and n-termini of peptides^29^. In half of the cases, the site annotation for gluconoylation was not correct, which can be explained by modified fragments not being observed in the peptide spectra due to its labile nature (denoted by # in Fig. 3B).

In addition to the biologically relevant post translational modifications, 15% of all peptides carried modifications classified as chemical modifications (Fig. 3C). Chemical modifications can for instance be introduced in the sample preparation process, such as over-alkylation of cysteines^30^ as well as oxidations on methionine, tryptophan or tyrosine residues induced in the sample preparation itself^31^ or in the peptide fragmentation process in the mass spectrometer^32,33^. We observed thousands of peptides with cysteine carbamidomethyl, which is introduced during the sample preparation by over alkylation^30^. We observed sodium and ammonium adducts, which can either come from solvents used in the laboratory or from the electrospray ionization^34,35^. Another chemical modification we observed was formylation from formic acid. Modification of phenylalanine at peptide n-termini can occur in the in-source fragmentation^36^ such as oxidation and dioxidation. We also found that aminoethylbenzenesulfon (AEBS) modifies many proteins by covalent attachment, predominantly on tyrosine residues and to a smaller extent on lysine and histidine residues. AEBS occurs due the use of protease inhibitors in the sample preparation.

**Figure 4:**
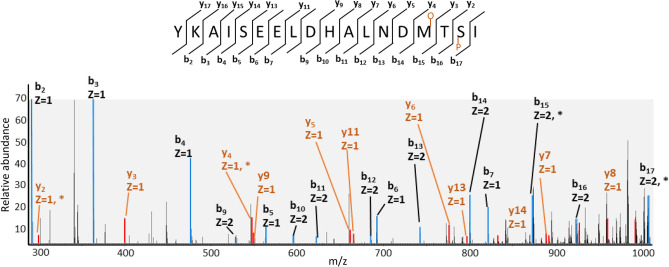
Fragmentation spectra of peptide with two PTMs: phosphorylation and oxidation. Highlighted peaks represent peptide fragments. b-ions show fragments from the peptide N-terminus side (blue) and y-ions show fragments from the peptide C-terminus (orange). Fragments with modifications are highlighted with *: b15, b17, y2 and y4. The peptide is oxidated on methionine and phosphorylated on serine. The inset figure at the top shows all fragment ions detected.

By applying the annotation tools PTM-sticker^11^ and PTM-Shepherd^37^ we were able to annotate the majority of the modification mass shifts identified and localized with a high accuracy. However, for approximately 15% of the mass shifts, we could not assign a known modification from the Unimod^38^ database. For the cases where the identified mass shift was absent in Unimod, PTM-Sticker created possible combinations of well-known modifications in Unimod and localized the amino acids of those combinations (Fig. 3D). One interesting case we observed was of a Δ Mass 95.961, which matched a combination of phosphorylation and oxidation. We verified these cases by manually checking the spectra and could localize both modifications on their corresponding amino acids (Fig. 4).

### PTMs and cardiac specific proteins

**Figure 5:**
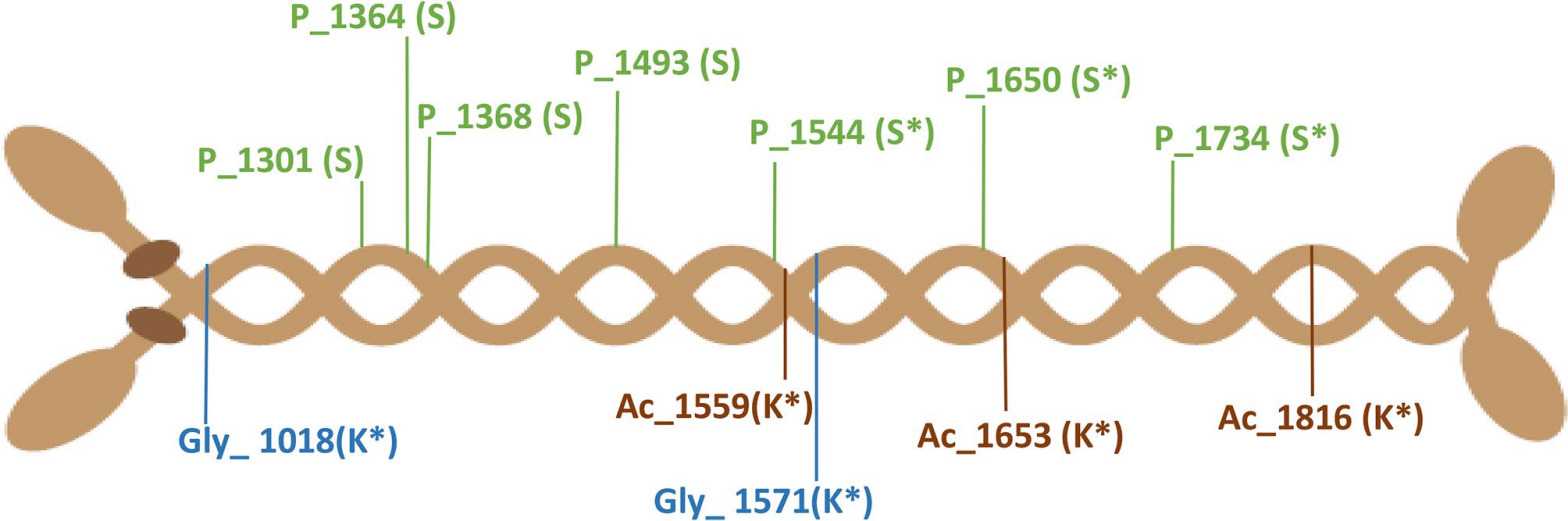
Cartoon representation of Myh6 and some of its PTMs. Three of the PTMs identified on Myh6 are illustrated in the cartoon: acetylation (Ac), phosphorylation (P) and glycosylation (Gly). The site and amino acid harbouring the modification is indicated. Sites with * represent novel sites. For a full list of modifications on Myh6, see Supplementary Table S6.

From the outcome of our analysis it is evident that there are many more post translational modifications in human hearts than previously described. To mention a few specific examples we observed that many myosin proteins were nitrosylated, e.g. the proteins encoded by *Myl4*, *Myh4*, *Myh6*, *Myl3*, *Myh3*, *Mybpc3* and *Tpm1*. Many of these proteins were also methylated, such as proteins encoded by *Myl9, Myh11, Myh7, Myl3, Myh6, Myh8, Tba3c, Tpm1, Tpm2, Tpm3, Ctna2* and *Desm*. To illustrate that the PTMs we report herein are also localized to cardiac specific proteins we here discuss modifications for a few proteins, where their dysfunction are linked to cardiac disease states. The selected proteins are merely representative examples to illustrate how the map we generated can be used for hypothesis generation of novel regulations of key proteins. As example proteins we highlight the cardiac proteins myosin heavy chain-α (*MYH6*), myosin heavy chain-β (*MYH7*), phospholamban (*PLN*), troponin I (*TNNI3*), myosin binding protein C3 (*MYBPC3*), voltage-gated sodium channel (*SCN5A*), ryanodine receptor 2 (*RYR2*) and the L-type voltage-gated calcium channel (*CACNA1C*). We detected these proteins with several modifications covering well known modifications, such as phosphorylation and acetylation and less studied modifications such as nitrosylation, methylation and di- and tri-oxidation. We also detected larger modifications on some of the proteins, such as glycosylation and ubiquitination. All modifications for these essential cardiac proteins are reported in Supplementary Table S6, where details for each chamber is included. For *MYH6*, methylation and oxidation were the two most frequent PTMs. However, we also identified multiple serine phosphorylation sites as well as acetylation and glycosylation on lysine residues (Fig. 5). We cross referenced the sites on *MYH6* using the PhosphoSitePlus^39^ resource. Most of the phosphorylation sites we identified have been reported previously, but we also identified novel phosphorylation sites, which we manually validated in the MS/MS spectrum (Supplementary Fig. S2). Similarly we identified known and novel sites on MYH6 for lysine acetylation.

The examples above illustrate that cardiac proteins are decorated with PTMs. When the impact of a PTM on protein function is evaluated, PTM stoichiometry changes between two conditions are of primary interest. The resource we have generated is merely a map of PTMs. We observed that protein phosphorylation, acetylation, kynurenine and methylation are all abundant PTMs in the human heart, and we thus set up a separate search focused on those PTMs to evaluate for differences in their abundances across the three cardiac chambers. We evaluated for significant differences by volcano plot analyses (Supplementary Fig. S3) and provide the corresponding data in a table format (Supplementary Table S7). From these analyses it is evident that there are site specific abundance differences between the two atrial chambers for all four PTMs (20 phosphorylation sites, 3 acetylation sites, 40 kynurenine sites, 9 methylation sites, Supplementary Fig. S3), as well as site-specific differences between left atrial and ventricular samples (64 phosphorylation sites, 19 acetylation sites, 84 kynurenine sites, 31 methylation sites, Supplementary Fig. S3). For instance, we find these phosphorylation sites to be significantly more abundant in left ventricle than in left atria: PKP2_pS82, PKP2_pS135, TTN_pS767, MYH7_pS1412, DSP_pS2608. We highlight these sites as they reside on proteins with essential functions in ventricular cardiomyocytes. Alike, we find significantly greater abundance of methylation in the left ventricle on the transcription factor BTF3_mK2 and the zink finger protein ZNF624_mK38. Lysine acetylation is more abundant in the ventricle on transcription factor SOX5_acK452 and mitochondrial proteins TIMM13_acK16 and IDH2_acK180.

The examples showcased here illustrate the level and variation of decorations of cardiac proteins by post translational modifications. The open search strategy we applied enabled us to assign PTMs to specific amino acids on specific proteins. We illustrate how this information can be utilized to investigate for quantitative differences in different heart regions in a separate analysis. Comprehensive lists of all PTMs identified in human heart samples are provided in the supplementary tables.

## Discussion

To fully understand the molecular mechanisms contributing to cardiac regulation, we need to expand our knowledge of post translational modifications affecting protein function in the heart. We know of PTMs that are crucial regulators of cardiac function and that are directly linked to cardiac diseases. However, we have only just begun to explore the complex landscape of PTMs in the heart. Herein we present data of approximately 150 different protein modifications measured in human heart tissues, which we identified by applying a suit of open search algorithms to broadly identify MS-detectable PTMs. The data was acquired from seven individuals and the PTMs were mapped to proteins across three of the cardiac chambers. For all PTMs we have assigned the modification to a specific amino acid on a specific protein. We show that the basal distribution of PTMs and affected amino acids is similar across cardiac chambers. Outlining PTMs for proteins involved in heart functions aid our ability to investigate the relation between protein chemistry and function. Novel PTMs that contribute to cardiac mechanism is expected in the comprehensive list of cardiac PTMs that we present herein.

We have focused our analyses on biological modifications, as they are the most relevant in context of cardiac function. However, we also identified several chemical modifications. For methodological and technical reasons, measured peptides may contain chemical modifications that need to be considered when evaluating acquired MS spectra. These chemical modifications for instance count small impurities in reagents used and reactions in the mass spectrometer itself. In the dataset presented herein, we provide details on many chemical modifications that are likely to be present in processed human heart samples as well as an explanation for their presence. From a biological point of view these modifications are of limited interest. However, chemical modifications are important to monitor to ascertain a consistent and high extent of spectra annotations throughout an experiment.

The scientific community has only just begun the exploration of molecular regulations in cardiac physiology and disease states queried by high-resolution mass spectrometry. Herein we present a global map of the PTMs present in human hearts, much beyond what is currently known. This data serves as a data-rich source for hypothesis generation in terms of which PTMs would be sensible to analyse in a quantitative manner evaluating their regulation in the heart –in physiological conditions as well as in disease states. That is, our data provides clues to novel PTMs involved in heart function that are yet to be studied in a functional context. Our results comprises a large map of characterized and classified PTMs in human hearts, which will serve as a resource to the scientific community interested in cardiac protein regulation.

## Methods

### Tissue collection and deep proteome measurements

We based this study on a published dataset, where all raw data are publically available^16^. For clarity, a brief description of the experimental conditions of the study by Linscheid and colleagues are provided below. Cardiac biopsy samples were collected from seven males undergoing mitral valve replacement surgery. Biopsies were collected from three cardiac chambers (LA, RA, and LV) and immediately frozen. Due to the nature of the surgical procedure the right ventricle was inaccessible and therefore not included in the study. Frozen tissue biopsies were homogenized, protein was digested in-solution by endoproteinase Lys-C and trypsin. 100 μg of the generated peptides was fractionated by micro-flow reverse-phase ultrahigh pressure liquid chromatograpy and collected in 12 concatenated fractions. Fractionated peptide samples were analysed by online reversed-phase liquid chromatography coupled to a Q-Exactive HF quadrupole Orbitrap tandem mass spectrometer. Full-MS spectra (375–1500 m/*z*) were acquired after accumulation of 3,000,000 ions in the Orbitrap (maximum fill time of 25 ms) at 120,000 resolution. A data-dependent Top12 method then sequentially isolated the most intense precursor ions (up to 12 per full scan) for higher-energy collisional dissociation (HCD) in an octopole collision cell. MS/MS spectra of fragment ions were recorded at resolution of 30,000 after accumulation of 100,000 ions in the Orbitrap (maximum fill time of 45 ms).

### Data analysis

#### Peptide identification using Comet-PTM database search

All the 252 Thermo raw files obtained from MS measurements were first converted into mzml format (according to guideline of Proteomic standard initiative) using RawConverter^40^. These converted raw files were then searched using Comet-PTM^11^ against a human protein fasta file from uniprot (downloaded on 15th of august 2019 with isoforms). For the search parameter a standard parameter file was used as described in the Comet-PTM user manual; precursor mass tolerance and fragment mass tolerance was set to 200 Daltons and 0.02 Daltons respectively. Precursor tolerance type and isotope error were set to 1. Precursor charge range was 2–4, maximum precursor charge 5 and maximum fragment charge 3. Only y- and b-ions were used for scoring. In addition trypsin digestion with 1 missed cleavages and fixed cysteine carbamidomethylation (57.021464 Da) was specified. Comet-PTM was developed to take the full advantages of multi-thread processing and to obtain fast results we used ‘Computerome’, a Danish national supercomputer for life sciences.

### Post-processing of PTM identification and FDR control

We used another tool SHIFTS^11^(Systematic Hypothesis-free Identification of modifications with controlled FDR based on ultra-Tolerant database Search) for post processing. SHIFTS is a program that identifies peaks in the Δ Mass distribution, assigns PSM to peaks and calculates FDR for peptide identification. Text Output from Comet-PTM was used an input for SHIFTS. For the peak picking modeling bin of 0.001 and identification score of 0.20 for mass error calculations was used. Using these parameter SHIFTS performs a mass recalibration and identifies all the PTM peaks and assign peptides to peaks. A FDR of 5% was used to report non-modified and PTM peptides.

### PTM annotation for identification

To annotate the identified Δ Mass we used an accompanying tool of SHIFTS called PTM-Sticker^11^. PTM-Sticker searches Δ Mass values against the Unimod database, taking into account the amino acid modified according to Comet-PTM output and also the preceding and consecutive residues, comparing them with the list of amino acids that could be subjected to the modification according to Unimod. If no amino acid was matched, the modification was considered as unassigned. All the results of PTM-Sticker are reported in the Supplementary Tables S2, S3 and S4. In addition we used PTM-Shepherd^37^ to confirm the PTM annotation from PTM-Sticker and to add additional information on the unknown masses.

### Evaluation of peptide identification performance by Comet-PTM and a conventional closed search

To evaluate the peptide identification performance of Comet-PTM compared to that of a conventional search engine we performed a search using Comet-PTM in a closed search mode. For the closed search, a subset of the dataset was analyzed. Specifically, four raw files were evaluated. The search was performed against the same protein sequence database used in this study. We included five variable modifications: oxidation (methionine), phosphorylation (serine, threonine, and tyrosine), acetylation (lysine), methylation (lysine, arginine) and kynurenine (tryptophan). For identification, peptide mass tolerance was set to 40 ppm and fragment mass tolerance was set to 0.02 Da. An FDR cut-off of 0.01 was applied and identified peptide scores were compared with the corresponding scores from the Comet-PTM search.

### Quantification of PTMs and comparative analysis between the cardiac chambers

To quantify potential abundance differences in PTM modified peptides across the different cardiac chambers, we searched the whole dataset using MaxQuant^41^. MaxQuant is open source software coupled with Andromeda search engine with the quantification modules included within the framework. In the parameters we defined phosphorylation (serine, threonine, and tyrosine), acetylation (lysine), methylation (lysine, arginine) and kynurenine (tryptophan) as variable modifications. MaxQuant provides separate output files for each PTM specified as a variable modification, which contain information on intensities of modified peptides along with PTM localization. Median normalization on log2-transformed intensities was performed using an in-house python script. The small number of missing values were imputed a using downshift imputation method where values were drawn from a distribution with width 0.3 and downshifted 1.8 compared to the original sample distribution. Differential PTM peptide expression was calculated using R package LIMMA based on empirical Bayes moderated test statistics^42^ for each of the PTMs included in the search. Peptides were considered significant at a *P* value < 0.05 and a fold-change greater than 2.

## Supplementary Information

Supplementary Information.

Supplementary Table 1.

Supplementary Table 2.

Supplementary Table 3.

Supplementary Table 4.

Supplementary Table 5.

Supplementary Table 6.

Supplementary Table 7.

## Data Availability

All raw data from MS measurements are publically available from previously published article^16^ at the PRIDE repository with the identifier PXD008722.
