# Supplementary material for: Proteome-wide profiling and mapping of post translational modifications in human hearts
Source: Sci Rep. 2021 Jan 26;11:2184. doi: 10.1038/s41598-021-81986-y (PMC7838296; doi:10.1038/s41598-021-81986-y)
Supplement: Supplementary file 1 — Supplementary Information. [file 41598_2021_81986_MOESM1_ESM.docx]

**Proteome-wide profiling and mapping of post translational modifications in human hearts**

Navratan Bagwan^1^, Henrik H. El Ali ^1^, and Alicia Lundby^1, 2 *^

Supplementary Material

# Contents

[Supplementary Figure S1 2](#_Toc59042304)

[Supplementary Figure S2 2](#_Toc59042305)

[Supplementary Figure S3 5](#_Toc59042306)

[Legends for Supplementary Tables 7](#_Toc59042307)

# Supplementary Figure S1

# Supplementary Figure S2

# Supplementary Figure S3

# Legends for Supplementary Tables

**Supplementary Table S1:** Summary table of all PTMs identified in the human heart samples covering three heart chambers. The table contains detailed descriptions of each modification as well as their frequency across the three chambers. The modifications are classified into categories.

**Supplementary Table S2:** List of all PTMs identified on proteins in left atrium samples. For each PTM, protein name, gene name, peptide sequence and information on modified amino acid is included.

**Supplementary Table S3:** List of all PTMs identified on proteins in left ventricle samples. For each PTM, protein name, gene name, peptide sequence and information on modified amino acid is included.

**Supplementary Table S4:** List of all PTMs identified on proteins in right atrium samples. For each PTM, protein name, gene name, peptide sequence and information on modified amino acid is included.

**Supplementary Table S5:** Expanded information on PTMs included in Figure 2.

**Supplementary Table S6:** List of PTMs identified in the left atrium, left ventricle and right atrium for specified cardiac proteins. The table contains protein name, gene name and peptide sequence information showing the site of modification.

**Supplementary Table S7:** List of quantified PTM containing peptides for four PTMs (kynurenin, phosphorylation, methylation and acetylation) across the three cardiac chambers.
